# Autistic Individuals’ Categorical Induction Abilities Improve by Mid-Adolescence

**DOI:** 10.1007/s10803-025-07082-6

**Published:** 2025-10-30

**Authors:** Grace Corrigan, Letitia R. Naigles

**Affiliations:** 1 Department of Communicative Sciences and Disorders, Michigan State University, 1026 Red Cedar Road, East Lansing, MI 48824, USA; 2 Department of Psychological Sciences, University of Connecticut, 406 Babbidge Road, Unit 1020, Storrs, CT 06269, USA

**Keywords:** Autism spectrum disorder, Categorical induction, Language, Adolescence

## Abstract

**Purpose:**

Categorical induction, the ability to transfer a known category’s properties to a new category member, develops early and robustly in typically developing (TD) children but has proven challenging for autistic children and adolescents. We investigated whether autistic and TD adolescents might perform similarly on a categorical induction task when expressive language was controlled for, and whether autistic and TD children’s categorical induction abilities improved longitudinally.

**Methods:**

Twenty-two TD and 20 autistic participants completed a ‘Diversity’ categorical induction task as adolescents. For the longitudinal analysis, we examined a subset (19 TD and 14 autistic participants) who had also completed an ‘Early’ categorical induction task as five-to-seven-year-olds.

**Results:**

When expressive language was controlled for, groups did not statistically differ on categorical induction performance in adolescence. Expressive language also predicted performance above and beyond nonverbal IQ in both groups. In the longitudinal analysis, we observed that both groups’ categorical induction performance significantly improved over time, and the magnitude of improvement did not differ by group.

**Conclusions:**

Contrary to previous literature on this subject, our findings suggest that categorical induction is not out of reach for autistic individuals; accounting for differences in expressive language between groups, autistic adolescents’ categorical induction performance mirrored that of their TD peers. Furthermore, our longitudinal analysis clarifies that the same autistic individuals who previously struggled with categorical induction could become more consistent.

## Introduction

The ability to categorize is a critically important part of life. Rapid assignment of a new entity to a category saves us time and cognitive labor: if we know what category the item belongs to, then we might know how it behaves or what traits it has. Research on categorization has revealed that typically developing (TD) children and adults form prototypes (Church et al., 2010; Gastgeb et al., 2012; Klinger & Dawson, 2001) and assign new items to existing categories (Ellawadi et al., 2016; Gastgeb et al., 2006) more consistently than children and adults on the autism spectrum.^1,2^ Categorical induction, which refers to the act of extending properties from a known category onto a new category member (Gelman & Markman, 1986; Gutheil & Gelman, 1997), has been less thoroughly studied with autistic participants, but has thus far demonstrated challenges for children and young adolescents on the spectrum (Naigles et al., 2013; Tecoulesco et al., 2021; see Wimmer et al., 2024 for a recent meta-analysis). Developmental improvements in categorical induction are frequently reported in TD samples (e.g., Rhodes & Liebenson, 2015); however, it is unclear whether older adolescents or young adults with autism spectrum disorder (ASD) would continue to display inconsistent categorical induction or become more consistent. Moreover, neither TD nor ASD literatures have investigated in depth why these developmental changes might occur: in particular, the degree to which they are rooted in developing nonverbal cognition or more advanced linguistic knowledge. The current study aims to fill these gaps.

### Strategies of Categorical Induction

While novel-item category learning and categorization tasks consistently pose challenges for autistic individuals, a recent meta-analysis has documented relatively small effect sizes and somewhat of a publication bias (Wimmer et al., 2024) and suggests that future research focus more on the moderator variables that might illuminate the heterogeneity observed within autism. We follow this suggestion with a somewhat different task, namely, categorical induction, which targets how—and which—properties extend within a given category. That is, categorical induction allows us to assume that new category members share some of the traits of known category members. A developmentally early key to unlocking category membership judgments is labeling (Gelman & Coley, 1990; Gelman et al., 1994); that is, hearing a label generally prompts two-to-five-year-old TD children to consider a new labeled item to be part of an already-labeled natural kind category, and not (necessarily) to be part of other categories (Gelman & Markman, 1986, 1987). Even TD children vary in this ability, however: in a longitudinal study, Tecoulesco et al. (2021) demonstrated that five-year-olds with more consistent categorical induction performance had shown stronger word learning biases when they were three years of age.

Adults rely on more than category labels for categorical induction, though. For example, various entities that all belong to the same overarching category (e.g., bears) may still differ in some of their properties (e.g., some bears are carnivores, and some are omnivores). Osherson et al. (1990) proposed that adults rely on cues such as sample size and diversity to help them decide whether to extend properties to new instances. *Sample size* refers to the amount of evidence being considered, where a larger set (e.g., grizzly bears, polar bears, and brown bears love onions) provides stronger evidence for a conclusion (e.g., black bears love onions) than a smaller set (e.g., grizzly bears and polar bears love onions). The more entities that share a given property, the more likely it is that another entity will share that property. *Diversity* refers to the similarity of entities to each other, such that those that differ more (e.g., lions and giraffes use norepinephrine as a neurotransmitter) constitute stronger evidence for a conclusion (e.g., rabbits use norepinephrine as a neurotransmitter) than those that are similar (e.g., lions and tigers use norepinephrine as a neurotransmitter). The more different the entities are (e.g., lions and giraffes, as opposed to lions and tigers) that share a given property, the more likely it is that the property can extend to another different entity (e.g., rabbits; Osherson et al., 1990).

Gutheil and Gelman (1997) demonstrated that neurotypical adults correctly extended more often than chance when diverse sets of entities were contrasted with homogenous sets (DH), and when larger sets, be they diverse or homogenous, were contrasted with single entities (DS, HS). In contrast, nine-year-olds performed at chance with both diversity and sample size contrasts (Gutheil & Gelman, 1997). Subsequent developmental research focused primarily on the diversity premise, and largely confirmed, in cross-sectional studies, that TD five-to-eight-year-olds generalize equivalently from diverse and non-diverse samples whereas nine-to-13-year-olds demonstrate increasing reliance on diversity-based reasoning (Li et al., 2009; Naigles et al., 2013; Rhodes et al., 2008; Rhodes et al., 2010; Rhodes & Liebenson, 2015). However, while variability is evident during development, few attempts have been made to distinguish or explain why some children begin using the diversity cue earlier than others. Our research seeks to address this gap, both with TD children and those diagnosed with autism.

### Categorical Induction in Autism

Categorical induction has also been studied in individuals diagnosed with ASD. Autism is characterized specifically by social communication differences and the presence of restricted or repetitive interests and behaviors (American Psychiatric Association, 2013), but many individuals on the spectrum present with additional challenges. A considerable body of research has shown that autistic individuals struggle with categorization in general (Beck et al., 2022; Ellawadi et al., 2016; Gastgeb et al., 2006; Klinger & Dawson, 2001; Nader et al., 2022; Van Overwalle et al., 2025), as well as with categorical induction in particular (Kelley et al., 2006; Naigles et al., 2013; Tecoulesco et al., 2021). Using a basic categorical induction task with labeled targets (Gelman & Markman, 1987), Tecoulesco et al., (2021) found that six-and-a-half-year-old children on the autism spectrum did not extend properties to category matches at rates different from chance, while five-and-a-half-year-old TD children extended to the category match significantly more consistently. Using diversity-based reasoning tasks, Naigles et al. (2013) assessed groups of autistic 10- and 13-year-olds with age-appropriate language skills. The 10-year-olds did not extend more often than chance on a simplified diversity task, whereas the 13-year-olds, tested on the DS and DH trials from Gutheil and Gelman (1997), selected the properties of the diverse samples at rates above chance (though still significantly less consistently than the TD 13-year-olds). Given the same tasks, their TD peers selected these properties at above-chance levels at both ages. The TD groups differed primarily from the autistic groups in their consistency of performance: most strikingly, at both ages approximately three-quarters of TD participants performed at Perfect (100%) or Almost Perfect (80%+) levels, with only about half of participants on the autism spectrum performing similarly.

### The Roles of Age and Diagnosis: Gaps in the Literature and the Current Studies

What are the factors that enable autistic or non-autistic individuals to perform consistently and/or above chance on categorization and categorical induction tasks? Four recent studies have focused mainly on nonlinguistic abilities and strategies. For example, Beck et al. (2022) reported that autistic adults relied on different features for category learning than neurotypical adults, Van Overwalle et al. (2025) found that lower accuracy in autistic individuals’ generalizing to novel stimuli was correlated with their increased intolerance to uncertainty, Ellawadi et al. (2016) demonstrated that autistic children’s lower accuracy with somewhat atypical items correlated with their nonverbal cognition, and Nader et al. (2022) observed better generalizations in autistic children who received simultaneously-presented stimuli rather than high-intensity feedback. Even so, Wimmer et al.’s (2024) recent meta-analysis calls for increased investigation of potential moderators or predictors of autistic individuals’ categorization abilities.

For the diversity/sample size cues to categorical induction that are of relevance here, chronological age emerges as a prime candidate; in the TD literature, younger children engage in diversity-based reasoning less consistently than children older than nine years of age or adults (Gutheil & Gelman, 1997; Naigles et al., 2013; Rhodes & Liebenson, 2015). But how is age influencing the ability to consider sample diversity when making categorical inductions? Here, the literature is much less clear. Some studies with school-age TD children do observe some diversity sensitivity when the task is massively simplified (Brandone, 2017; Rhodes & Liebenson, 2015); however, this literature generally does not report other characteristics of the children, which might provide clues as to what facilitates more advanced categorical induction. Development with age can lead to more advanced conceptual structures as well as more advanced linguistic structures (Edwards et al., 2022), either/both of which might enable children to consider sample size and sample diversity when drawing categorical inductions. Within the literature involving children and adolescents on the autism spectrum, factors including nonverbal cognition, vocabulary, and pragmatics have been shown to positively correlate with diversity-based reasoning (Naigles et al., 2013); however, these variables have not always been compared in the same model. Moreover, the role of autism diagnosis is also unclear, because the TD participants who outperformed age-matched autistic participants in Naigles et al. (2013) had language levels two to three years ahead of their chronological ages, whereas the autistic participants had age-appropriate language levels. Thus, differences by diagnosis might actually be ‘hidden’ by language and/or nonverbal cognition differences.

Based on these gaps in the literature, we developed three research questions:

1. Though the TD categorical induction literature has included adults, categorical induction has not been investigated in autistic individuals older than 13 years of age. Because autism is associated with delayed language and cognitive development (Tager-Flusberg et al., 2005), it may be the case that autistic individuals need more time to ‘catch up’ to TD comparison groups. Would an older adolescent group of autistic participants show similar performance to TD participants?
2. Do language and/or nonverbal cognition correlate with categorical induction performance in autistic or TD adolescents, and how do they compare in the same model?
3. Cross-sectional studies of categorization and categorical induction have been the norm; however, these studies do not rigorously address whether or not children’s categorical induction abilities actually develop over time. Would the same children tested at multiple timepoints show improved categorical induction performance, and which early factors might predict improvement?

In the current study, we address Questions 1 and 2 via a cross-sectional analysis of TD and autistic teenagers and young adults who completed the full Gutheil and Gelman (1997) task (hereafter, the ‘Diversity’ task). Though these participants are not matched on language, they are matched on chronological age and are older than any other TD and autistic children studied thus far in this field, which may be sufficient to elicit similar group performance. Additionally, these TD participants are a more normative sample, cognitively and linguistically, than the TD participants in Naigles et al. (2013). Moreover, we rigorously examine the relationships between concurrent language, nonverbal IQ (NVIQ), and categorical induction within-groups. To address Question 3, we incorporate longitudinal data collected from many of these participants and compare these autistic and TD children’s performance on the easier Gelman and Markman (1987) task (hereafter, the ‘Early’ task) as five-to-seven-year-olds to their performance on the Diversity task as teenagers. Our unique ability to examine the same children’s categorical induction at two timepoints will provide insight into whether individual children can improve their categorical induction consistency over time. We additionally consider the potential predictive role of early linguistic and cognitive variables for longitudinal changes in categorical induction development.

## Method

### Participants

Participants were a subset of the Longitudinal Study of Early Language (LSEL; Naigles & Fein, 2017), tested about 15 years after study onset (“Onset”). Children on the spectrum were initially recruited, through ASD service providers in the New England area, to be within six months of their diagnosis and using little to no phrase speech. TD children were recruited locally, via birth announcements, flyers, and community word of mouth, to have similar spoken language characteristics to the autism sample. Consequently, at the Onset visit, the TD children averaged 1.5 years of age and the children on the spectrum averaged one year older. Every participant’s diagnostic status was confirmed via administration of the Autism Diagnostic Observation Schedule-Generic (ADOS-G; Lord et al., 1999) and the two groups were matched on expressive language, operationalized as raw scores on the expressive language subscale of the Mullen Scales of Early Learning (MSEL; Mullen, 1995). Details of our participants at their Onset visit are presented in Table 1.

For our investigation of Questions 1 and 2, we conducted a categorical induction task at the most recent of the study’s visits (“Outcome”). The subset who was seen at the Outcome visit included 24 TD adolescents and 23 autistic adolescents; however, two TD adolescents were excluded due to loss of data, and three autistic adolescents were excluded due to inability to understand and complete the task. Thus, the final sample included 22 TD adolescents (six girls, 16 boys) and 20 autistic adolescents (three girls, 17 boys). Due to the timing of the Outcome visit’s data collection, the groups did not differ statistically in age, though the ASD group was numerically older. Moreover, they now differed in language as well as NVIQ, and continued to differ in autism symptomatology, as shown in Table 2.

For our investigation of Question 3, we examined a smaller LSEL subset who completed not only the Outcome visit’s Diversity categorical induction task, but also the Early categorical induction task at a previous (“Intermediate”) visit (see Tecoulesco et al., 2021, for cross-sectional findings). Details about the makeup of this smaller, longitudinally tracked group can be found in Supplementary Materials (Part I, Tables S1, S2, S3).

### Overall Procedure

Testing was conducted in the participants’ homes at all visits. Measures at each timepoint were administered in two sessions, at most two weeks apart.

### Materials: Standardized Test Measures

Participants’ language, NVIQ, and autism symptomatology were assessed with age-appropriate measures at all timepoints. For details about each measure, see Table 3.

### Materials: Categorical Induction Tasks

#### Diversity Categorical Induction Task

The Diversity task (Gutheil & Gelman, 1997) was introduced with the following story:

I went to the zoo last week and I have some pictures of the animals I saw there. I know about some of the animals because the man who works at the zoo told me about them, but I don’t know about a lot of the other animals. I’m going to show you these pictures and you can help me answer some questions about them. Now remember, the questions are still going to be about things we can’t see in the pictures, but might still be there.

A total of nine sets of animals were shown to participants (detailed item descriptions presented in Supplementary Materials, Part II, Table S4). In the three DH trials, participants saw five photographs of different-looking (‘diverse’) animals in a category (e.g., whales) and five photographs of similar-looking (‘homogenous’) animals in the same category. In the three DS and three HS trials, participants saw a diverse group or homogenous group, respectively, contrasted with another photograph of a single animal. The target animal for all trials was from the same category but looked different from all of the animals presented so far in that trial. The script for a HS trial would be as follows:

These snakes [point to homogenous group of five snakes] have an orange spot on their tails. This snake [point to single snake] has a red spot on its tail. Here’s another snake [present target image]. Do you think this snake has an orange spot on its tail like these snakes [point back to homogenous group] or a red spot on its tail like this snake [point back to single snake]?

Blocks were presented in two orders, randomized across participants: DH-HS-DS-HS-DH-DS-DH-DS-HS (Table S4) or the reverse. Performance on the task did not differ based on presentation order (*t*(37) = 1.03, *p* =.309). Participants could answer verbally or by pointing to the set of animals they believed shared a trait with the target animal. Participants received one point for each choice of the most inclusive option: the diverse group in DH and DS trials, and the homogenous group in HS trials.

#### Early Categorical Induction Task

In the Early task (Gelman & Markman, 1987), participants were shown colored pencil drawings of a total of eight natural kinds: four animate (e.g., a snake) and four inanimate (e.g., a chunk of salt). In each of the eight blocks, the participant was told the item’s name and a property it held. Next, four other drawings were presented: (1) an “identical” match featuring the same item in a different position; (2) a category match, which belonged to the same category but looked different; (3) a perceptual match, which looked similar to the initial item but belonged to a different category; and (4) a distractor, which shared neither the initial item’s category nor appearance. The category match was the item of interest, as it assessed whether participants would extend the property to a shared category member regardless of its appearance. The script for a given block was as follows:

This brown rabbit (point to picture of brown rabbit; Target) eats grass. Does this brown rabbit (point to picture of another brown rabbit in a different position; Identical Match) also eat grass? Does this white rabbit (point to picture of white rabbit; Category Match) also eat grass? Does this long-eared squirrel (point to picture of long-eared brown squirrel; Perceptual Match) also eat grass? Does this lizard (point to picture of lizard; Distractor) also eat grass?

Participants received one point for every correct extension of the property to the Category match, and zero points if they did not extend the property to the Category match. Summaries of the stimuli are presented in Supplementary Materials (Part II, Table S5).

### Analysis Plan

Thirty-nine participants completed the Diversity task at the Outcome visit. Three additional participants (all in the ASD group) attempted this task but did not complete it; however, they were able to complete the Early task, and so their scores on that task were included. Every member of the longitudinal subset, seen at both the Intermediate and Outcome visits, completed the Early task at the Intermediate visit and the Diversity task at the Outcome visit. To analyze categorical induction performance regardless of task, scores on both tasks were converted to percent correct. Subsequent analyses were also conducted without the three Early task completers (Supplementary Materials; Part III, Tables S6 and S7).

To address Questions 1 and 2, we analyzed the scores from the Outcome visit. One-sample t-tests were used to determine whether each group performed above chance (50%). An Analysis of Covariance (ANCOVA) was used to determine whether the ASD and TD groups’ categorical induction performance differed when controlling for concurrent language, which differed between groups (Table 2). Within-group linear regressions were then conducted with Outcome visit NVIQ (Model 1) and expressive language (Model 2) measures as predictors, and categorical induction performance as the dependent variable. Following Naigles et al. (2013), participants in each group were then further classified by how consistently they extended (i.e., how consistently they were able to transfer the property of the more inclusive set of animals onto the target animal in the Diversity task, or how consistently they were able to transfer the target property onto the Category match in the Early task). Participants were classified as “Perfect” (100% of trials), “Almost Perfect” (87.50–88.89%), “Consistent” (62.50–77.78.50.78% of trials), “Moderate” (50.00–55.56.00.56% of trials), or “Non-Extender” (< 50.00%). A Chi-square test of independence was used to determine whether the count of each extender type differed by group. Finally, we conducted an exploratory analysis of performance by item type (DH, DS, HS), which included only participants who completed the Diversity task (22 TD, 17 autistic). A 2 × 3 mixed ANCOVA (group x item type, controlling for concurrent language) was conducted. Mauchly’s Test of Sphericity indicated that the assumption of sphericity had been violated (Χ^2^(2) = 6.98, *p* =.031); therefore, degrees of freedom were corrected using the Huynh-Feldt estimates of sphericity (ε = 0.93).

To address Question 3, we compared the scores from the longitudinal subset who was tested at the Intermediate and Outcome visits. A 2 × 2 (group x visit) mixed ANOVA was used to investigate main effects of group and visit and a group*visit interaction. Additionally, the number of participants who improved (had higher scores on the Diversity task compared with the Early task) was calculated and compared for each group. Within-group linear regressions were then conducted with Intermediate visit NVIQ (Model 1) and receptive language (Model 2) measures as predictors, and difference score (percent correct at the Intermediate visit subtracted from percent correct at the Outcome visit) as the dependent variable.^3^

Data visualization in R (v4.4.0) was performed using the *tidyverse* (v2.0.0; Wickham et al., 2019), *ggplot2* (v3.4.4; Wickham 2016), *ggforce* (v0.4.2; Pedersen, 2022), and *lme4* (v1.1–35.5.5; Bates et al., 2015) packages.

## Results

Q1: Do autistic adolescents show similar categorical induction performance to TD adolescents?

Q2: Do concurrent language and/or NVIQ predict categorical induction performance within groups, and how do these predictors compare in the same model?

**Fig. 1:**
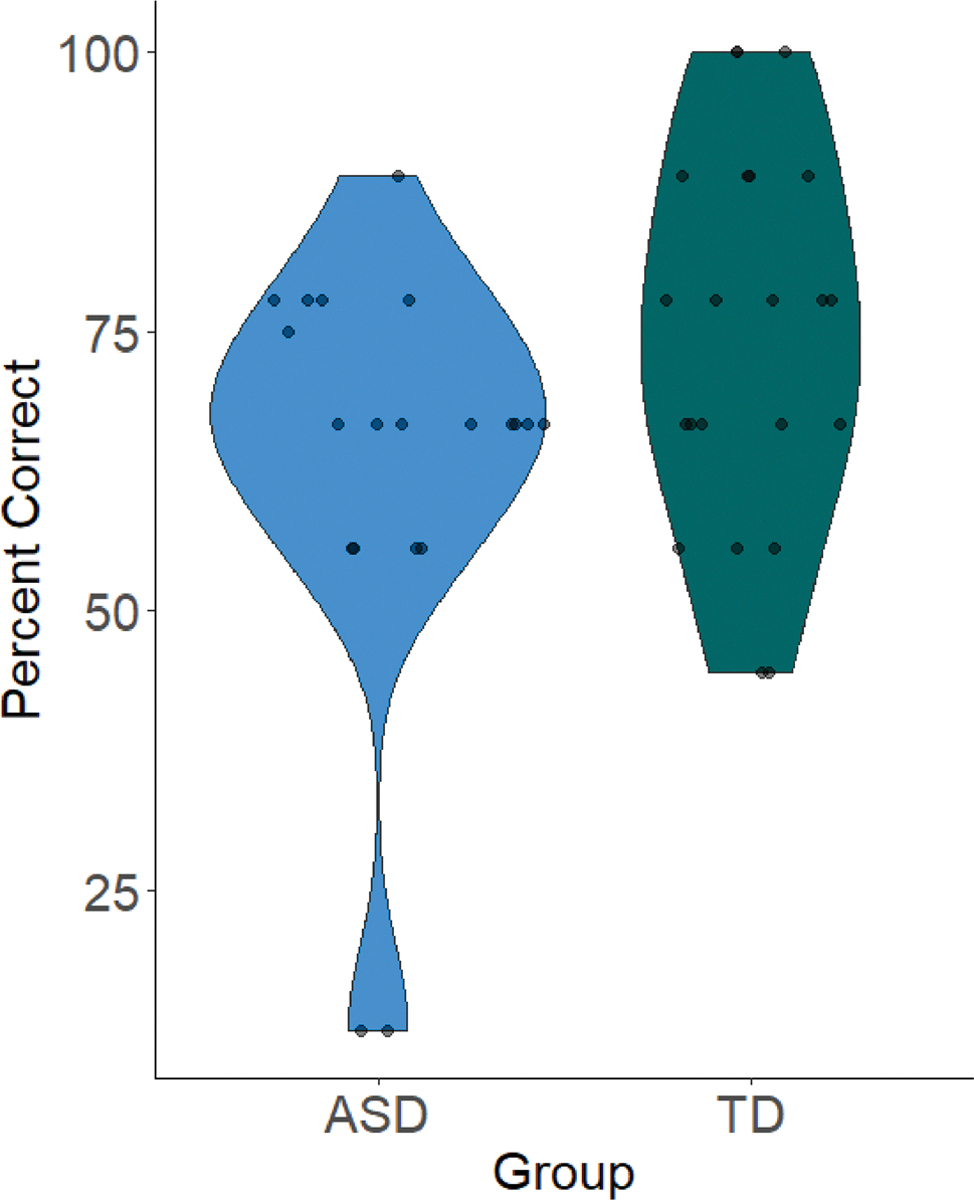
Violin plot showing percent correct (y-axis) by group (ASD and TD; x-axis). Dots represent individual cases. Percent correct did not differ by group

One-sample t-tests revealed that the TD group (*M* = 74.24%, *SD* = 16.93%) performed significantly better than chance (*t*(21) = 6.72, *p* <.001, Cohen’s *d* = 1.43), as did the ASD group (*M* = 62.78%, *SD* = 19.35%; *t*(19) = 2.76, *p* =.008, Cohen’s *d* = 0.66). The ANCOVA controlling for concurrent language (CELF-5 summed raw scores) revealed no significant group difference in categorical induction performance (*F*(1,39) = 0.24, *p* =.629; see Fig. 1).^4^

Within-group regressions (see Table 4) revealed that for the TD group, NVIQ was a significant positive predictor, such that every 1-point increase in NVIQ predicted a categorical induction score increase of 0.56. Further, the predictive utility of the model significantly increased with the addition of expressive language, *F*-change (1,19) = 5.62, *p* =.028. This second model predicted 38.8% of the variance in categorical induction performance. When including both expressive language and NVIQ in the model, NVIQ was no longer a significant predictor whereas expressive language remained a significant positive predictor, such that every 1-point increase in expressive language predicted a categorical induction score increase of 0.69. For the ASD group, NVIQ was also a significant positive predictor, such that every 1-point increase in NVIQ predicted a categorical induction score increase of 0.45. Further, the predictive utility of the model significantly increased with the addition of expressive language, *F*-change(1,17) = 5.93, *p* =.026. This second model predicted 48.4% of the variance in categorical induction performance. When including both expressive language and NVIQ in the model, NVIQ was no longer a significant predictor whereas expressive language remained a significant positive predictor, such that every 1-point increase in expressive language predicted a categorical induction score increase of 0.73.

**Fig. 2:**
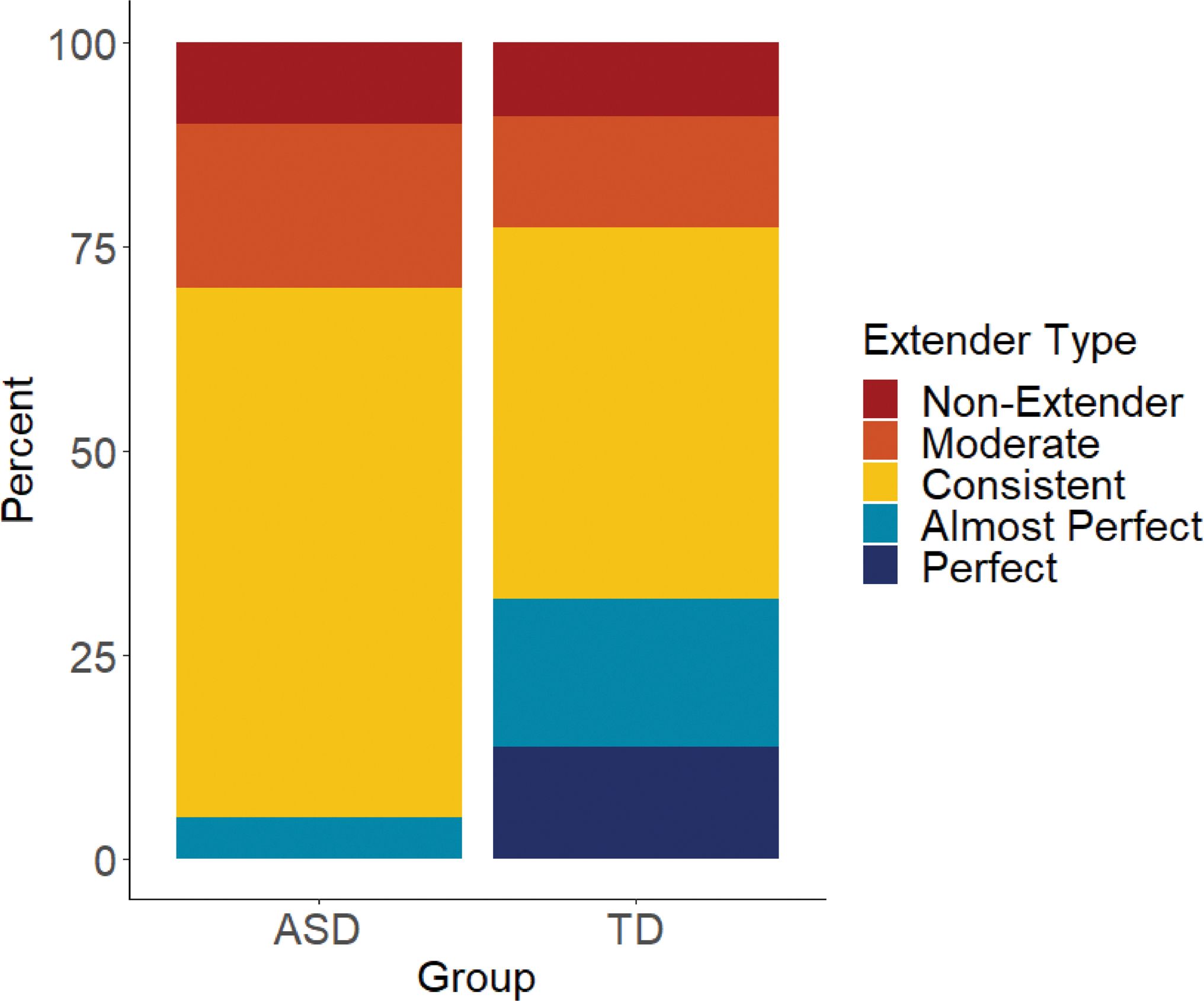
Stacked bar chart showing the percentage (y-axis) of each group (ASD and TD; x-axis) who belonged to each Extender Type category (top to bottom: Non-Extender, Moderate, Consistent, Almost Perfect, Perfect). Groups did not differ on Extender Type distribution

Participants in each group were then classified into one of five groups (Perfect, Almost Perfect, Consistent, Moderate, Non-Extender) according to their extension consistency (see Supplementary Materials, Part IV, Table S8). For both groups, Consistent performance was the modal response. A Chi-Square test of independence revealed no significant group differences in the distribution of extender types (Χ^2^(4) = 5.25, *p* =.263; see Fig. 2).^5^

**Fig. 3:**
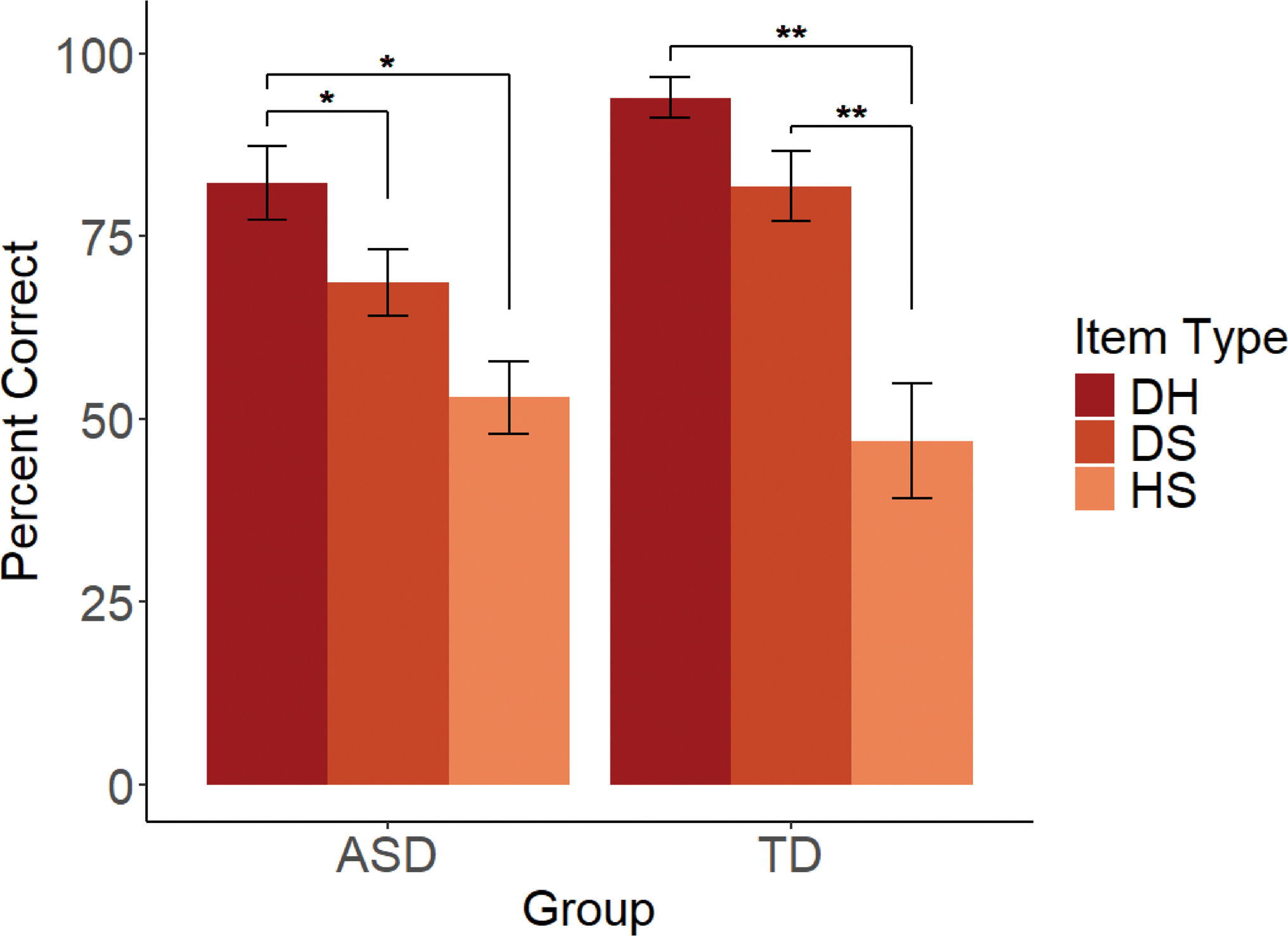
Clustered bar chart showing percent correct (y-axis) by group (ASD and TD; x-axis) and item type (left to right: DH, DS, HS). *p <.01. **p <.001. Error bars show standard errors

The mixed ANCOVA for our exploratory analysis of performance by item type (DH, DS, HS) revealed a main effect of item type (*F*(1.87,67.22) = 4.60, *p* =.015, *η*^2^ = 0.11), such that scores on DH items significantly exceeded scores on DS items (*p* =.004), and scores on both DH and DS items significantly exceeded scores on HS items (*p*s < 0.001). As expected, there was no main effect of group on Diversity task scores (*p* =.814). However, a group*item type interaction emerged, *F*(1.87,67.22) = 3.67, *p* =.033, *η*^2^ = 0.09. For both groups, scores on DH items significantly exceeded scores on HS items (*p*s < 0.008). For autistic participants, scores on DH items were also significantly higher than scores on DS items (*p* =.009), and scores on DS and HS items did not differ (*p* = 1.000). TD participants exhibited a different response pattern: scores on DS items significantly exceeded scores on HS items (*p* <.001), but scores on DH and DS items did not differ (*p* =.825; see Fig. 3).^6^

Q3: Does autistic and TD participants’ categorical induction performance improve over time, and which early factors might predict improvement?

**Fig. 4:**
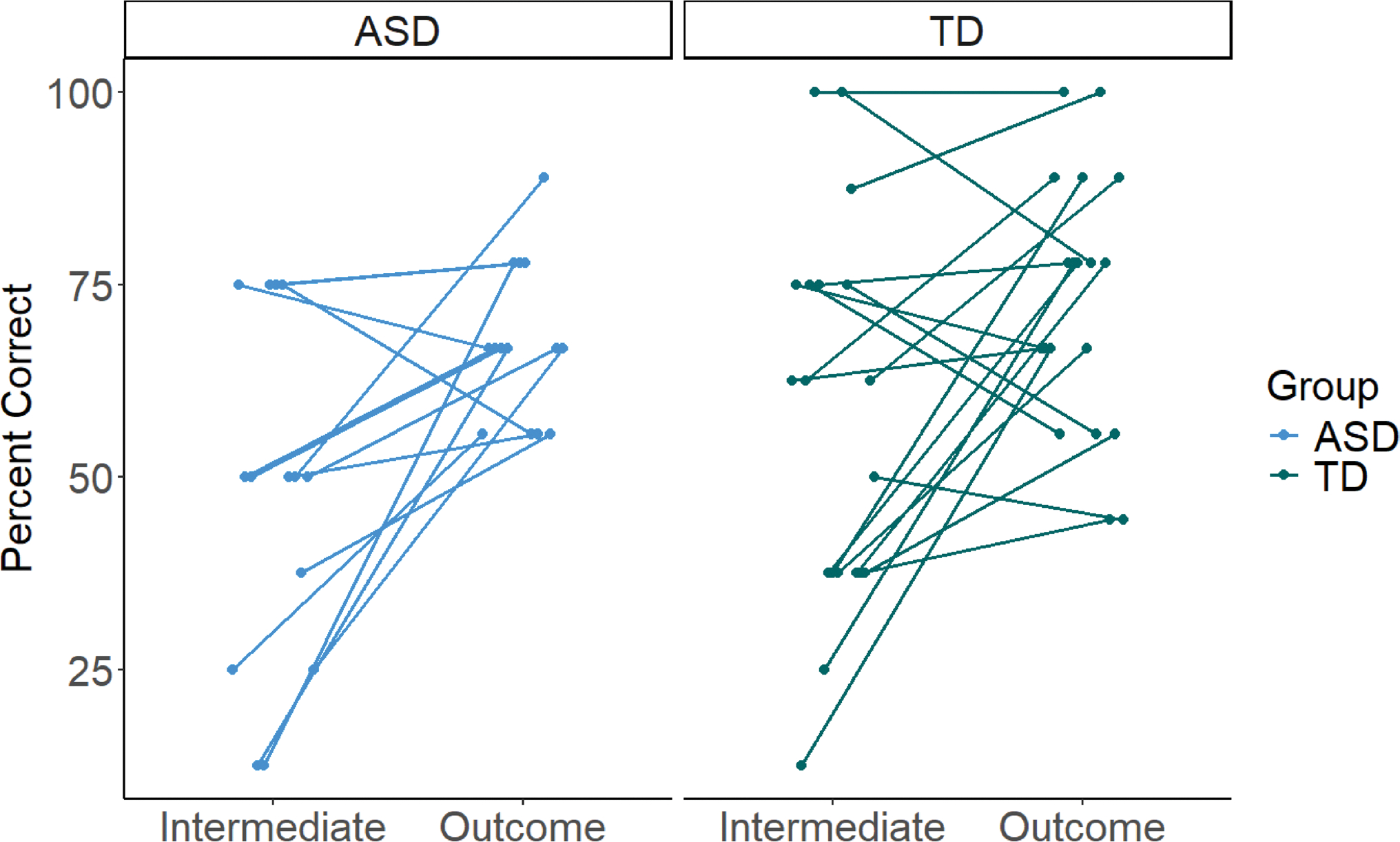
Scatterplot showing longitudinal change in percent correct (y-axis) from the Intermediate to Outcome visit (x-axis). Each case is represented by two dots (one at each timepoint) connected by a line. The ASD group is on the left and the TD group is on the right. At the Intermediate visit, percent correct was calculated as the percentage of correct extensions of the target property to the Category Match, out of eight trials. At the Outcome visit, percent correct was calculated as the percentage of correct (more inclusive option) choices, out of nine trials

The 2 × 2 (group x visit) mixed ANOVA revealed a main effect of visit (*F*(1,31) = 16.79, *p* <.001, *η*^2^ = 0.35), such that across groups, categorical induction performance improved significantly from the Intermediate to the Outcome visit. Notably, no main effect of group emerged (*F*(1,31) = 1.87, *p* =.182), indicating that across visits, the ASD and TD groups’ performance did not differ. Further, no visit*group interaction emerged (*F*(1,31) = 0.32, *p* =.578), suggesting that there was no statistical difference in the magnitude of the groups’ improvement over time.^7^ The longitudinal changes in categorical induction performance are presented pictorially in Fig. 4.

Individually, 12 autistic participants (85.71%) and 13 TD participants (68.42%; including one participant who achieved perfect performance at both visits) improved from the Intermediate to the Outcome visit. A Chi-Square test of independence revealed no significant group differences in the number of participants who improved (Χ^2^(1) = 1.31, *p* =.252).

Within-group regressions revealed that for the TD group, NVIQ at the Intermediate visit significantly positively predicted difference scores. When receptive language was added, neither NVIQ nor receptive language at the Intermediate visit remained significant predictors of difference scores (results described further in Supplementary Materials, Part V, Table S9). For the ASD group, neither NVIQ nor receptive language were significant predictors (Table S9).

## Discussion

In this research we asked three questions: (1) Would *older adolescent* autistic individuals, unlike five-to-12-year-olds, show consistent ability to extend the properties of categories to new instances, on par with TD peers? (2) Would nonverbal cognition, language, and/or the two in combination predict task performance in either or both groups? (3) Would our use of a longitudinal dataset allow us to demonstrate longitudinal improvement in categorical induction, expanding upon the cross-sectional differences usually reported? Our findings provided affirmative answers to all three questions.

Our cross-sectional analysis demonstrated that by 17 years of age, these autistic teenagers performed well above chance on a sample size and diversity categorical induction task. More importantly, they achieved similar performance to non-autistic teenagers in categorical induction tasks when expressive language—which varied between groups—was controlled for. The modal response for both groups was Consistent Extender, further illustrating the similarity in group performance on these tasks. Moreover, within each group, expressive language ability proved to be the strongest predictor of categorical induction performance, over and above nonverbal IQ. Longitudinally, we saw that both groups significantly improved from the Intermediate to the Outcome visit, and the magnitude of task improvement from the Intermediate to Outcome visit did not differ by group. Interestingly, receptive language at the Intermediate visit did not predict the teens’ degree of improvement by the Outcome visit.

Taken together, these findings indicate that category analysis is not out of reach for autistic individuals. Though literature on this topic has largely shown inconsistent categorization performance in autistic children, we found that autistic teenagers performed comparably to TD teenagers. The fact that our TD teens comprise a sample whose language levels are close to their chronological age, rather than two or more years ahead, suggests that previous findings of group differences in categorical induction (e.g., Naigles et al., 2013) may have been better attributable to language differences.

Our results thus underscore the role of concurrent language in categorical induction. Controlling for expressive language in TD and autistic participants whose language differed was sufficient to negate group differences in task performance. Furthermore, expressive language positively predicted task performance in each group, nullifying the initially significant predictive role of NVIQ. Thus, linguistic knowledge showed itself to be a dominant contributor to the use of sample size and diversity premises/strategies in categorical induction. How might language play out this role? One possibility is that language provides ways to talk about these premises, bringing them to conscious awareness. For example, our participants may have said to themselves, *If only one snake has this trait*, *it could be unique to that snake…but if these snakes that look so different all have this trait*, *then it could be something spread across all snakes.* While the TD literature has pointed to growth in conceptual knowledge as the basis for increased usage of sample diversity (Rhodes & Liebenson, 2015), we note that the school years coincide with increased metalinguistic ability (Hoff, 2013), and that possibly it is this explicit use of language in reasoning tasks that explains not only our findings but also those in the TD literature. However, we did not ask our participants to describe their cognitive processes during the task, so we cannot say for certain whether they employed their metalinguistic abilities in this way. Future studies may benefit from exploring this possibility.

We further speculate that students who are more advanced linguistically may also receive more advanced schooling in, for example, the kind of biology curriculum that would support the use of sample size and diversity to make inductions about natural kind categories. We did not collect data concerning the educational experiences of the participants; however, we did administer the Vineland Adaptive Behavior Scales-Third Edition (Vineland-3; Sparrow et al., 2016) to their caregivers. The Vineland-3 Written Language subscale includes items asking if the children read at the 2nd, 4th, 6th, or 9th grade levels. Of the 14 autistic participants tracked longitudinally, eight read at the 9th grade level or above, four read at the 6th grade level but not at the 9th grade level, and two, including those with the lowest categorical induction scores, read at the 4th grade level but not at the 6th grade level. We speculate that one of the factors that enabled the autistic teens to improve their categorical induction abilities may have been academic experiences in biology—and possibly other areas—that are consistent with higher reading levels.

Our finding that concurrent language is the strongest correlate of categorical induction performance in both autistic and non-autistic adolescents further extends the general categorization literature, which has focused primarily on nonlinguistic features that may support novel category learning (e.g., Beck et al., 2022; Van Overwalle et al., 2025). Moreover, our findings somewhat diverge from those of Ellawadi et al. (2016), who reported linguistic correlates to TD 5-year-olds’ performance with atypical instances in a categorization task, but non-linguistic correlates (NVIQ) to the performance of 7-year-olds on the autism spectrum with the same task. These differing findings might be attributable to the delayed language development of children diagnosed with autism, further supporting the importance of longitudinal investigations. In sum, we concur with Wimmer et al.’s (2024) suggestion that future studies of all categorization-relevant knowledge focus on the attributes that might help assist those on the spectrum to achieve this knowledge, and we further encourage such investigations to cast a wide net in selecting these attributes.

Finally, the single difference between the TD and ASD groups as adolescents involved the ordering of performance on the DH, DS, and HS trials. Whereas both the TD and ASD groups extended most consistently on DH trials, and least consistently on the HS trials, they differed in how the DS trials clustered. For the TD group, the DH and DS trials did not differ significantly from each other, while both differed from the HS trials. Essentially, the TD group prioritized diversity; diverse samples were consistently weighted more heavily than non-diverse samples, and trials that only differed in sample size yielded poorer performance. In contrast, for the ASD group, the DS and HS trials did not differ significantly from each other, while both differed from the DH trials. Autistic participants seemed to rely on sample diversity only when sample size was not a consideration; trials that involved sample size, regardless of diversity information status, yielded similar performance. Thus, the TD group relied on diversity more often than the ASD group, but overall, both groups were less consistent with the sample size premise. Interestingly, this ordering—and stronger reliance on diversity than sample size—is contrary to the predictions of Osherson et al. (1990), although consistent with the findings of Naigles et al. (2013), and suggests a possible developmental pattern in how these two premises emerge in children’s inductive reasoning.

### Limitations and Future Directions

We note several limitations to our study design, which may impact interpretation of the results. First, though our use of the same Diversity task items (snakes, birds, etc.) as Gutheil and Gelman (1997) lends credence to our extension of their findings, it also poses some limitations. Our analysis of differential performance on DH, DS, and HS trials was confounded by stimulus type; for example, we cannot say with certainty whether participants performed least consistently on the HS snake item because of the HS comparison or because snakes were more difficult for some reason. Future iterations of the Diversity task should include DH, DS, and HS items that all involve the same animal or other natural kind.

Second, our longitudinal examination of categorical induction performance is a strength; however, it was limited by the administration of different categorical induction tasks at each timepoint (the Early task at the Intermediate visit; the Diversity task at the Outcome visit). Though both the Early and Diversity tasks tap categorical induction, they require different induction strategies. The Early task involves no more sophisticated reasoning than attention to category labels, but the Diversity task necessitates consideration of both sample size and diversity, more advanced concepts. In both groups, most children who performed poorly at the Intermediate visit showed large improvements, while children who were above chance at the Intermediate visit plateaued or improved only slightly (see Fig. 4 for case-by-case change in performance). Thus, although we observed stronger performance in both groups on a more difficult categorical induction task at the Outcome visit, relative to their performance on an easier categorical induction task at the Intermediate visit, we cannot definitively conclude that the exact same categorical induction strategies improved over time. Future longitudinal research should consider administering the Early and Diversity tasks at both timepoints to track the development of specific categorical induction strategies.

Additionally, and possibly related to this last point, we were unable to identify measures at the Intermediate visit that predicted the degree of change/improvement in categorical induction in the ASD group at the Outcome visit. The absence of significant relationships could not be attributable to the lack of variability of performance at the Outcome visit, because concurrent language level was associated with Diversity task performance in adolescence. Instead, this absence could be because of the (on average) 10-year span between the Intermediate and Outcome visits, and because we had little data concerning the children’s education and intervention experiences during that span. We encourage future research to consider these experiences (e.g., Thomas et al., 2023) in more detail as possible drivers of growth in categorical induction understanding.

Finally, we recognize that our sample sizes, particularly for the longitudinal analysis, were quite small, which may also have impacted the predictive strength of Intermediate visit measures. As is the nature of longitudinal research, we experienced considerable attrition in both the TD and ASD groups over the course of a decade, resulting in a much smaller subset of participants who could be included in the longitudinal analysis. It is sensible to recommend some degree of caution in interpreting the longitudinal findings for this reason; however, our main effect of visit did reach significance at *p* <.001, providing substantial support for our assertion that both groups improved over time.

## Conclusion

The ability to extend known categorical properties to new members is fundamental to navigating the complexities of the world. Rapid categorical induction saves both time and cognitive effort by facilitating understanding of a novel item based on its category membership. Young individuals on the autism spectrum appear to have difficulty with this ability; however, by their teenage years, many of them, especially those with more advanced language, have achieved significant mastery. We conclude that that not only are autistic and non-autistic teenagers well-equipped to complete categorical induction tasks, but that the very same participants who struggle with categorical induction in childhood can improve over the course of a decade.

## Supplementary Material

SM for Corrigan & Naigles 2025

**Supplementary Information** The online version contains supplementary material available at https://doi.org/10.1007/s10803-025-07082-6.

## Figures and Tables

**Table 1:** Participant characteristics at the Onset visit

	TD (*N* = 22)	ASD (*N* = 20)			

	*M (SD)*	*M (SD)*	*F*	*p*	ŋ^2^
Age (months)	**18.86 (1.49)**	**32.25 (6.16)**	**97.73**	**< 0.001**	**0.71**
MSEL Expressive Language	18.18 (5.49)	19.30 (6.54)	0.36	0.551	–
MSEL Receptive Language	22.95 (3.33)	23.65 (8.20)	0.13	0.716	–
MSEL Visual Reception	24.59 (4.16)	26.80 (5.32)	2.27	0.140	–
ADOS-G total	**1.23 (1.60)**	**15.25 (3.84)**	**246.99**	**< 0.001**	**0.86**

*MSEL* Mullen Scales of Early Learning, *ADOS-G* Autism Diagnostic Observation Schedule-Generic. All scores are raw scores unless otherwise specified. Bolding indicates a significant group difference at *p* = .05

**Table 2:** Participant characteristics at the Outcome visit

	TD (*N*=22)	ASD (*N*=20)			

	*M (SD)*	*M (SD)*	*F*	*p*	ŋ^2^
Age (years)	16.32 (2.51)	17.17 (3.28)	0.90	0.349	–
CELF-5 total	**207.64 (19.80)**	**141.70 (65.46)**	**20.32**	**< 0.001**	**0.34**
CELF-5 LMI (std. score)	**105.14 (13.52)**	**77.80 (20.33)**	**26.77**	**< 0.001**	**0.40**
*N* in LMI normal range (≥ 86)	20	8	–	–	–
DAS-II total	**66.50 (14.01)**	**49.75 (15.96)**	**13.11**	**< 0.001**	**0.25**
DAS-II SNC (std. score)	**106.00 (13.72)**	**80.50 (23.85)**	**18.46**	**< 0.001**	**0.32**
*N* in SNC normal range (≥ 85)	21	7	–	–	–
ADOS-2 total	**2.67 (3.10)**	**13.35 (7.04)**	**40.24**	**< 0.001**	**0.51**

*CELF-5* Clinical Evaluation of Language Fundamentals-5th Edition, *LMI*Language Memory Index, *DAS-II* Differential Ability Scales-2nd Edition, *SNC* Special Nonverbal Composite, ADOS-2 Autism Diagnostic Observation Schedule-2nd Edition. All scores are raw scores unless otherwise specified. Bolding indicates a significant group difference at *p* = .05. One TD participant is missing ADOS-2 data

**Table 3:** Assessments administered at each visit

Assessment	Description	Subtests

Onset Visit		

MSEL (Mullen, 1995)	NVIQ and expressive/receptive language assessment	Expressive Language, Receptive Language, *and* Visual Reception
Onset & Intermediate Visit		
ADOS-G (Lord et al., 1999)	Semi-structured autism assessment	Module 1, Module 2, *or* Module 3
Intermediate Visit		
TACL-3 (Carrow-Woolfolk, 1999)	Receptive language assessment	Vocabulary, Grammatical Morphemes, *and* Elaborated Phrases and Sentences
DAS (Elliott, 1990)	NVIQ assessment	Preschool Record Form *or* School-Age Record Form
Intermediate & Outcome Visit		
ADOS-2 (Lord et al., 2012)	Semi-structured autism assessment	Module 3 *or* Module 4
Outcome Visit		
CELF-5 (Wiig et al., 2013)	Expressive/receptive language assessment	Word Classes, Following Directions, Recalling Sentences, Formulated Sentences, Sentence Assembly, *and* Semantic Relationships
DAS-II (Elliott, 2007)	NVIQ assessment	Recall of Designs, Pattern Construction, Matrices, *and* Sequential and Quantitative Reasoning

*MSEL* Mullen Scales of Early Learning, *ADOS-G* Autism Diagnostic Observation Schedule-Generic, *TACL-3* Test for Auditory Comprehension of Language-3rd Edition, *DAS* Differential Ability Scales, *ADOS-2* Autism Diagnostic Observation Schedule-2nd Edition, *CELF-5* Clinical Evaluation of Language Fundamentals-5th Edition, *DAS-II* Differential Ability Scales-2nd Edition

**Table 4:** Hierarchical models predicting categorical induction performance by group

	Predictor Statistics					Model Statistics			

	*B*	*SE*	*β*	*t*	*P*	*R^2^*	*F*	*df*	*^P^*
*Model 1 (TD)*						0.21	5.24	1,20	0.033
Outcome IQ	0.56	0.25	0.46	2.29	0.033				
*Model 2 (TD)*						0.39	6.03	2,19	0.009
Outcome IQ	0.19	0.27	0.16	0.72	0.481				
Outcome Language	0.69	0.29	0.52	2.37	0.028				
*Model 1 (ASD)*						0.31	7.89	1,18	0.012
Outcome IQ	0.45	0.16	0.55	2.81	0.012				
*Model 2 (ASD)*						0.48	7.99	2,17	0.004
Outcome IQ	−0.04	0.25	−0.05	−0.16	0.873				
Outcome Language	0.73	0.30	0.74	2.43	0.026				

Outcome IQ = DAS-II Special Nonverbal Composite (standard score; Elliott, 2007); Outcome Language = CELF-5 Expressive Language Index (standard score; Wiig et al., 2013)

## Footnotes

1 We include identity-first (*autistic person*) and person-first (*person on the autism spectrum*) language throughout this paper to reflect the variations in preference expressed by the autistic community (Bottema-Beutel et al., 2021).

2 The first author identifies as neurodivergent; the second author identifies as neurotypical.

3 Regressions were also conducted with ‘difference score divided by Outcome-Intermediate age difference’ as the dependent variable; similar findings were obtained.

4 When concurrent language was not controlled for, the TD group’s percent correct was higher than the ASD group’s, *t*[40] = 2.05, *p* =.047, Cohen’s *d* = 0.63.

5 Within-group ordinal regressions were conducted to examine the relationships between extender type categorization and Outcome visit measures, with very similar findings to the above regressions investigating percent correct and Outcome measures.

6 There were no group differences within item type; the ASD group’s scores did not differ from the TD group’s scores on DH, HS, and DS items (ps>0.075).

7 When analyses were performed using z-scores instead of percent correct, similar findings were obtained.
