# Supplementary material for: Autistic Individuals’ Categorical Induction Abilities Improve by Mid-Adolescence
Source: J Autism Dev Disord. Author manuscript; Available in PMC 2025 Dec 15. (PMC12703915; doi:10.1007/s10803-025-07082-6)
Supplement: SM for Corrigan & Naigles 2025 [file NIHMS2123354-supplement-SM_for_Corrigan___Naigles_2025.docx]

Supplementary Materials for:

Autistic individuals’ categorical induction abilities improve by mid-adolescence

1. **Characteristics of the Longitudinal Subset at Each Visit**

Participants in the longitudinally tracked subset completed categorical induction tasks at two separate visits. The first (Intermediate) visit occurred when the TD children averaged 5 years of age and the children on the spectrum averaged 6 years of age. The Outcome visit, as described in Study 1, took place 10 years after the Intermediate visit on average. The participants who were seen at both the Intermediate and Outcome visits included 23 TD participants and 22 autistic participants; however, four TD participants were excluded due to loss of data at either visit, and eight participants on the spectrum were excluded due to inability to understand and complete the task at one or both visits. Thus, the final sample included 19 TD participants (five girls, 14 boys) and 14 participants on the spectrum (two girls, 12 boys). As with the broader cross-sectional sample, the groups in this subset were matched on expressive and receptive language at the Onset visit; however, unlike the entire sample, the ASD group in this subset had higher visual reception scores than the TD group at the Onset visit (Table S1).

**Table S1** Characteristics of the longitudinal subset at the Onset visit

|  | TD (*N* = 19) | ASD (*N* = 14) |  | | |
| --- | --- | --- | --- | --- | --- |
|  | *M (SD)* | *M (SD)* | *F* | *p* | η^2^ |
| Age (months) | **18.84 (1.50)** | **30.21 (5.51)** | **74.31** | **< .001** | **0.71** |
| MSEL Expressive Language | 17.53 (4.72) | 20.86 (6.38) | 2.98 | .094 | - |
| MSEL Receptive Language | 22.68 (3.11) | 25.79 (7.01) | 2.96 | .095 | - |
| MSEL Visual Reception | **24.05 (4.14)** | **27.57 (4.38)** | **5.54** | **.025** | **0.15** |
| ADOS-G total | **1.21 (1.55)** | **14.00 (3.46)** | **205.23** | **< .001** | **0.87** |

*Note*. MSEL = Mullen Scales of Early Learning; ADOS-G = Autism Diagnostic Observation Schedule-Generic; all scores are raw scores unless otherwise specified

At the Intermediate visit, the TD group had higher language scores than the ASD group, except for on the Vocabulary subtest of the TACL-3 (Table S2). Of the 33 longitudinally tracked participants, 24 (14 TD, 10 ASD) received the DAS at the Intermediate visit as their NVIQ measure. Three children in the ASD group completed the Preschool Record Form due to their lower language and cognitive level. Their standard scores are reported in Table S2 as DAS NC (Nonverbal Cluster), which is derived from three DAS subtests on the Preschool Record Form: Picture Similarities, Pattern Construction (Standard Administration), and Copying. All other participants who received the DAS completed the School-Age Record Form. Their standard scores are reported in Table S2 as DAS SNC (Special Nonverbal Composite), which is derived from four DAS subtests on the School-Age Record Form: Recall of Designs, Pattern Construction (Standard Administration), Matrices, and Sequential & Quantitative Reasoning.

**Table S2** Characteristics of the longitudinal subset at the Intermediate visit

|  | TD (*N* = 19) | ASD (*N* = 14) |  | | |
| --- | --- | --- | --- | --- | --- |
|  | *M (SD)* | *M (SD)* | *F* | *p* | η^2^ |
| Age (years) | **5.16 (0.78)** | **6.07 (0.56)** | **13.46** | **< .001** | **0.30** |
| TACL-3 Vocabulary | 35.11 (5.41) | 32.79 (6.62) | 1.23 | .277 | - |
| TACL-3 Grammatical Morphemes | **28.53 (7.81)** | **20.00 (9.55)** | **7.95** | **.008** | **0.20** |
| TACL-3 Elaborated Phrases & Sentences | **27.00 (8.21)** | **18.43 (11.35)** | **6.36** | **.017** | **0.17** |
| TACL-3 Quotient (std. score) | **120.58 (10.34)** | **90.79 (21.62)** | **27.74** | **< .001** | **0.47** |
| *N* in TACL-3 Quotient normal range (≥85) | 19 | 8 | - | - | - |
| DAS NC/SNC (std. score) | **104.71 (12.53)** | **85.70 (19.73)** | **8.37** | **.008** | **0.28** |
| *N* in DAS NC/SNC normal range (≥85) | 14 (/14) | 5 (/10) |  |  |  |
| ADOS-G total | **1.74 (2.00)** | **13.71 (7.02)** | **50.31** | **< .001** | **0.62** |

*Note*. TACL-3 = Test for Auditory Comprehension of Language-3^rd^ Edition; MSEL = Mullen Scales of Early Learning; DAS = Differential Ability Scales; NC = Nonverbal Cluster; SNC = Special Nonverbal Composite; ADOS-G = Autism Diagnostic Observation Schedule-Generic; all scores are raw scores unless otherwise specified

The other nine participants (five TD, four ASD) received the MSEL Visual Reception subscale (not reported in Table S2) as their NVIQ measure at the Intermediate visit because they were part of an earlier cohort who did not receive the DAS. Given the small sample size, a Mann-Whitney U test was used to measure whether groups differed in their visual reception score. The results indicated that for these nine participants, there was not a difference in the TD (*M(SD)* = 44.60(3.36)) and ASD (*M(SD)* = 47.00(3.16)) groups’ scores at the Intermediate visit, *U* = 5.50, *p* = .286.

By the Outcome visit, nearly 10 years later, the groups had further diverged on language, NVIQ, and autism symptomatology. Participants in the longitudinal subset showed the same pattern of group differences as the cross-sectional participants: the groups were the same chronological age, and the TD participants had significantly higher language and NVIQ and continued to have lower autism symptomatology than the autistic participants (Table S3).

**Table S3** Characteristics of the longitudinal subset at the Outcome visit

|  | TD (*N* = 19) | ASD (*N* = 14) |  | | |
| --- | --- | --- | --- | --- | --- |
|  | *M (SD)* | *M (SD)* | *F* | *p* | η^2^ |
| Age (years) | 16.25 (2.48) | 17.20 (3.34) | 0.89 | 0.354 | **-** |
| CELF-5 total | **204.53 (19.49)** | **163.86 (53.39)** | **9.42** | **.004** | **0.23** |
| CELF-5 LMI (std. score) | **102.63 (12.62)** | **84.29 (17.42)** | **12.34** | **.001** | **0.29** |
| *N* in LMI normal range (≥86) | 17 | 7 | - | - | - |
| DAS-II total | **64.47 (12.93)** | **51.36 (15.53)** | **7.00** | **.013** | **0.18** |
| DAS-II SNC (std. score) | **103.84 (12.98)** | **85.64 (20.97)** | **9.46** | **.004** | **0.23** |
| *N* in SNC normal range (≥85) | 18 | 7 | - | - | - |
| ADOS-2 total | **2.33 (2.38)** | **10.86 (5.86)** | **34.77** | **< .001** | **0.54** |

*Note.* CELF-5 = Clinical Evaluation of Language Fundamentals-5^th^ Edition; LMI = Language Memory Index; DAS-II = Differential Ability Scales-2^nd^ Edition; SNC = Special Nonverbal Composite; ADOS-2 = Autism Diagnostic Observation Schedule-2^nd^ Edition; all scores are raw scores unless otherwise specified. One TD child is missing ADOS-2 data

1. **Categorical Induction Task Stimuli Descriptions**

**Table S4** Diversity categorical induction task stimuli

| Animal type | First set (property) | Second set (property) | Trial type |
| --- | --- | --- | --- |
| Whales | Homogenous group (yellow spot in mouth) | **Diverse group (tan spot in mouth)** | DH |
| Monkeys | **Homogenous group (purple tongue)** | Single animal (blue tongue) | HS |
| Tuna fish | Single animal (blue spot in mouth) | **Diverse group (green spot in mouth)** | DS |
| Snakes | **Homogenous group (orange spot on tail)** | Single animal (red spot on tail) | HS |
| Butterflies | Homogenous group (blue eyes) | **Diverse group (gray eyes)** | DH |
| Birds | **Diverse group (orange spot on wing)** | Single animal (red spot on wing) | DS |
| Frogs | Homogenous group (croak all day) | **Diverse group (croak at end of day)** | DH |
| Turtles | **Diverse group (no speckles on stomach)** | Single animal (small speckles on stomach) | DS |
| Clownfish | Single animal (smooth scales) | **Homogenous group (rough scales)** | HS |

*Note.* Correct (more inclusive option) answers are bolded

**Table S5** Early categorical induction task stimuli

| Original | Property | Identical Match | Category Match | Perceptual Match | Distractor |
| --- | --- | --- | --- | --- | --- |
| Brown rabbit | Eats grass | Brown rabbit | White rabbit | Long-eared squirrel | Lizard |
| Small brown snake | Lays eggs | Small brown snake | Cobra | Small brown worm | Cow |
| Small blue bird | Feeds its babies mashed-up food | Small blue bird | Black bird | Blue butterfly | Dog |
| Cat with skunk marks | Can see in the dark | Cat with skunk marks | Brown cat | Skunk | Dinosaur |
| Chunk of salt | Melts snow | Chunk of salt | Fine-grained salt | Marble | Rock |
| Tan shell | Is smooth inside | Tan shell | Colorful conch shell | Tan stone | Metal |
| Yellow oil | Floats on water | Yellow oil | Brown oil | Yellow honey | Diamond |
| Dirty gold nugget | Melts in a hot oven | Dirty gold nugget | Gold bar | Clump of dirt | Chalk |

After presentation of the target item in each block, the participants were asked to repeat the information they had heard (e.g., “What is this? What can it do?”) to ensure understanding. The presentation of items within each block, and order of blocks, was the same for all participants. However, to avoid participants picking up on a pattern, the order of items varied between blocks, such that the Identical, Distractor, Category, and Perceptual items were presented in different orders in each block. Participants’ yes/no responses to the four questions in each block were recorded on a response sheet.

1. **Cross-Sectional Analyses Without Early Task Completers**

Analyses were redone excluding the three Early task completers to see if results differed when only Diversity task completers were included. The ASD group’s (*N* = 17) mean categorical induction performance was higher (*M(SD)* = 67.97(9.53)) without the three participants who completed the Early task at the Outcome visit. The ANCOVA controlling for concurrent language (CELF-5 summed raw scores) still revealed no significant group difference in categorical induction performance (*F*(1,36) = 0.06, *p* = .815).^[[1]](#footnote-1)^ Participants in each group were then classified into one of three groups according to their extension consistency on the Diversity task only (Table S6).

**Table S6** Type of extender by group

| TD (*N* = 22) | ASD (*N* = 17) | Type of Extender |
| --- | --- | --- |
| 7 (31.82%) | 1 (5.88%) | Almost Perfect / Perfect (87.50-100%) |
| 10 (45.45%) | 12 (70.59%) | Consistent / Proficient (62.50-77.78%) |
| 5 (22.73%) | 4 (23.53%) | Moderate (≤ 55.56%) |

A Chi-Square test of independence revealed no significant group differences in the distribution of extender types (Χ^2^(2) = 4.22, *p* = .121)

In the hierarchical linear regression examining the impact of NVIQ and expressive language on the smaller ASD group’s categorical induction performance, Model 1 was significant and predicted 34% of the variance in performance (Table S7). NVIQ was a significant positive predictor, such that every 1-point increase in NVIQ predicted a categorical induction score increase of 0.27. Although Model 2 was significant and predicted slightly more variance in categorical induction performance (36.4%), there was no significant increase in model utility, *F*-change(1,14) = 0.52, *p* = .482. Additionally, neither NVIQ nor expressive language were significant predictors of performance in Model 2.

**Table S7** Linear regression predicting categorical induction performance

|  | Predictor Statistics | | | | | Model Statistics | | | |
| --- | --- | --- | --- | --- | --- | --- | --- | --- | --- |
|  | *B* | *SE* | *β* | *t* | *p* | *R^2^* | *F* | *df* | *p* |
| Model 1 |  |  |  |  |  | 0.34 | 7.73 | 1,15 | .014 |
| Outcome IQ | 0.27 | 0.10 | 0.58 | 2.78 | .014 |  |  |  |  |
| Model 2 |  |  |  |  |  | 0.36 | 4.00 | 2,14 | .042 |
| Outcome IQ | 0.18 | 0.16 | 0.38 | 1.10 | .289 |  |  |  |  |
| Outcome Language | 0.14 | 0.20 | 0.25 | 0.72 | .482 |  |  |  |  |

*Note*. Outcome IQ = DAS-II Special Nonverbal Composite (standard score; Elliott, 2007); Outcome Language = CELF-5 Expressive Language Index (standard score; Wiig et al., 2013)

1. **Extender Type by Group for All Participants**

**Table S8** Type of extender by group

| TD (*N* = 22) | ASD (*N* = 20) | Type of Extender |
| --- | --- | --- |
| 3 (13.64%) | 0 (0.00%) | Perfect (100%) |
| 4 (18.18%) | 1 (5.00%) | Almost Perfect (87.50%-88.89%) |
| 10 (45.45%) | 13 (65.00%) | Consistent (62.50-77.78%) |
| 3 (13.64%) | 4 (20.00%) | Moderate (50.00-55.56%) |
| 2 (9.09%) | 2 (10.00%) | Non-Extender (< 50.00%) |

*Note.* Table includes the three Early task completers

1. **Regressions Predicting Change in Performance Among the Longitudinal Subset**

For the TD group, Model 1 was significant and predicted 30.7% of the variance in difference scores (percent correct at the Intermediate visit subtracted from percent correct at the Outcome visit; Table S9). NVIQ at the Intermediate visit was a significant positive predictor, such that every 1-point increase in NVIQ predicted a difference score increase of 1.04 (an increase of 1.04 from the Intermediate to the Outcome visit). Although Model 2 was significant and predicted slightly more variance in difference scores (32.4%), there was no significant increase in model utility, *F*-change(1,16) = 0.40, *p* = .536. Additionally, neither NVIQ nor receptive language at the Intermediate visit were significant predictors when both were included in Model 2.

**Table S9** Hierarchical models predicting difference scores by group

|  | Predictor Statistics | | | | | Model Statistics | | | | | |
| --- | --- | --- | --- | --- | --- | --- | --- | --- | --- | --- | --- |
|  | *B* | *SE* | *β* | *t* | *p* | *R^2^* | *F* | *df* | | *p* | |
| Model 1 (TD) |  |  |  |  |  | 0.31 | 7.52 | 1,17 | | .014 | |
| Intermediate IQ | 1.04 | 0.38 | 0.55 | 2.74 | .014 |  |  |  | |  | |
| Model 2 (TD) |  |  |  |  |  | 0.32 | 3.83 | 2,16 | | .044 | |
| Intermediate IQ | 0.87 | 0.48 | 0.46 | 1.82 | .087 |  |  |  | |  | |
| Intermediate Language | 0.39 | 0.61 | 0.16 | 0.63 | .536 |  |  |  | |  | |
| Model 1 (ASD) |  |  |  |  |  | 0.09 | 1.20 | 1,12 | .296 | |  |
| Intermediate IQ | 0.33 | 0.30 | 0.30 | 1.09 | .296 |  |  |  |  | |  |
| Model 2 (ASD) |  |  |  |  |  | 0.23 | 1.63 | 2,11 | .240 | |  |
| Intermediate IQ | -0.20 | 0.47 | -0.18 | -0.42 | .685 |  |  |  |  | |  |
| Intermediate Language | 0.67 | 0.48 | 0.61 | 1.40 | .188 |  |  |  |  | |  |

*Note.* Intermediate IQ = DAS Special Nonverbal Composite/Nonverbal Cluster (standard scores; Elliott, 1990) or MSEL Visual Reception (standard score; Mullen, 1995); Intermediate Language = TACL-3 Quotient (standard score; Carrow-Woolfolk, 1999)

For the ASD group, Model 1 was not significant, and NVIQ was not a significant predictor of difference scores (see Table S9, above). Model 2 was also not significant and there was no significant increase in model utility, *F*-change(1,11) = 1.97, *p* = .188. Accordingly, neither NVIQ nor receptive language at the Intermediate visit were significant predictors when both were included in Model 2.

1. When concurrent language was not controlled for, there was no group difference in performance, *t*[34.20] = 1.46, *p* = .153, Cohen’s *d* = 0.44. [↑](#footnote-ref-1)
